# A Late-Detected Paraganglioma in a Young Patient with Resistant Hypertension and Severe Aortic Regurgitation—A Case Report and Review of the Literature

**DOI:** 10.3390/jcm12144694

**Published:** 2023-07-14

**Authors:** Sabina Istratoaie, Emese Kovacs, Simona Manole, Andreea Ioana Inceu, Dan Damian Axente, Raluca Maria Bungărdean, Adela Mihaela Șerban

**Affiliations:** 1 Department of Pharmacology, Toxicology, and Clinical Pharmacology, ”Iuliu Haţieganu” University of Medicine and Pharmacy, 400337 Cluj-Napoca, Romania; 2 Department of Cardiology, ”Niculae Stăncioiu” Heart Institute, 400001 Cluj-Napoca, Romania; 3 Department of Radiology, “Niculae Stăncioiu” Heart Institute, 400001 Cluj-Napoca, Romania; 4 Department of Radiology, “Iuliu Hatieganu” University of Medicine and Pharmacy, 400012 Cluj-Napoca, Romania; 5 Cluj-Napoca Municipal Clinical Hospital, 400139 Cluj-Napoca, Romania; 6 Department of Pathology, ”Iuliu Hatieganu” University of Medicine and Pharmacy, 400012 Cluj-Napoca, Romania; 7 5th Department of Internal Medicine, ”Iuliu Haţieganu” University of Medicine and Pharmacy, 400012 Cluj-Napoca, Romania

**Keywords:** paraganglioma, aortic regurgitation, multimodality imaging, secondary arterial hypertension

## Abstract

Background: Paraganglioma is a rare neuroendocrine tumor derived from chromaffin cells. The overproduction of catecholamines accounts for the presenting symptoms and cardiovascular complications. The clinical presentation frequently overlaps with the associated cardiac diseases, delaying the diagnosis. Multimodality imaging and a multidisciplinary team are essential for the correct diagnosis and adequate clinical management. Case Summary: A 37-year-old woman with a personal medical history of long-standing arterial hypertension and radiofrequency ablation for atrioventricular nodal reentry tachycardia presented with progressive exertional dyspnea and elevated blood pressure values, despite a comprehensive pharmacological treatment with six antihypertensive drugs. The echocardiography showed a bicuspid aortic valve and severe aortic regurgitation. The computed tomography angiography revealed a retroperitoneal space-occupying solid lesion, with imaging characteristics suggestive of a paraganglioma. The multidisciplinary team concluded that tumor resection should be completed first, followed by an aortic valve replacement if necessary. The postoperative histopathology examination confirmed the diagnosis of paraganglioma. After the successful resection of the tumor, the patient was asymptomatic, and the intervention for aortic valve replacement was delayed. Discussion: This was a rare case of a late-detected paraganglioma in a young patient with resistant hypertension overlapping the clinical presentation and management of severe aortic regurgitation. A multimodality imaging approach including transthoracic and transesophageal echocardiography, computed tomography, and magnetic resonance imaging had an emerging role in establishing the diagnosis and in guiding patient management and follow-up. The resection of paraganglioma was essential for the optimal timing of surgical correction for severe aortic regurgitation. We further reviewed various cardiovascular complications induced by pheochromocytomas and paragangliomas.

## 1. Introduction

Pheochromocytomas and paragangliomas (PPGL) are rare catecholamines-secreting neuroendocrine tumors of the autonomic nervous system. Although closely related to pheochromocytoma in terms of clinical manifestation and histology, paraganglioma develops from chromaffin tissue in the extra-adrenal parasympathetic and sympathetic paraganglia, while pheochromocytoma arises from chromaffin tissue in the adrenal medulla. The release of catecholamines accounts for the classic triad of palpitations, headaches, and sweating, but the intermittent nature of this release delays the diagnosis [1]. PPGLs may manifest as cardiac complications in the initial presentation due to excessive catecholamine secretion or related to consequences of longstanding hypertension. PPGLs may lead to hypertensive crisis, arrhythmias, acute coronary syndrome, or catecholamine-induced dilated cardiomyopathy, overlapping clinical presentations that can pose diagnostic and management challenges [2].

The use of different imaging techniques is essential for the correct diagnosis and follow-up of PPGLs [3]. Contrast-enhanced computed tomography (CT) or T2-weighted magnetic resonance imaging (MRI) is recommended for tumor localization and to define the optimal approach to surgery. The entire retroperitoneum should be included in the standard imaging technique, because the extra-adrenal lesions are mainly situated in the retroperitoneum [1]. CT, rather than MRI, is recognized as the first choice imaging modality due to a better spatial resolution of the involved anatomical areas, providing details about the nearby anatomical structures. MRI is recommended for the examination of metastatic PPGLs, paragangliomas arising in the skull base or neck, and for patients allergic to the CT contrast or patients that need limitations to radiation exposure [4].

Multimodality imaging is essential for the assessment of the AR mechanism, regarding aortic valve morphology and thoracic aorta dimensions, severity, and hemodynamic consequences [5]. Although transthoracic echocardiography is the standard imagistic method for AR evaluation, in case of inconclusive findings, transesophageal echocardiography improves the evaluation of AR severity and mechanism [5]. Cardiac magnetic resonance (CMR), the current reference standard for the measurement of the left ventricle mass, volume, and systolic function, has the potential to provide important diagnostic and prognostic information for patients with AR [6]. CT is the method of choice for an accurate evaluation of the thoracic aorta and to rule out coronary artery disease, particularly in patients that undergo surgical replacement of the aortic valve [5].

We report an undiagnosed paraganglioma in a young woman with resistant hypertension who presented with severe AR. Multimodality imaging using echocardiography, CT angiography, and MRI and prompt multidisciplinary care were required to attain an accurate diagnosis and to establish the right course of clinical management. The paraganglioma resection was crucial for the optimal timing of intervention for aortic regurgitation, as it changed the course of her clinical management and ultimately enabled a good cardiac outcome. A comprehensive analysis of various reported cardiovascular complications of PPGLs is further presented.

## 2. Case Presentation

A 37-year-old woman was admitted to the hospital due to progressive exertional dyspnea for three months. She had an 18-year history of arterial hypertension and underwent radiofrequency ablation for atrioventricular nodal reentry tachycardia (AVNRT) 13 years ago. At that time, antihypertensive treatment was introduced, and a suspicion of secondary hypertension was raised, but no adrenal investigation was performed. Despite the progressive increase of antihypertensive therapy, her blood pressure (BP) remained uncontrolled under the maximal dose of a beta blocker, an angiotensin II receptor antagonist, a calcium channel blocker, thiazide diuretic, an alpha-1 adrenergic receptor antagonist, centrally active drugs, and a mineralocorticoid receptor antagonist, the highest recorded BP levels being 220/120 mmHg. The patient denied a family history of heart disease and relevant personal history such as smoking, alcohol use, or drug consumption.

On presentation, the patient was hemodynamically stable, her blood pressure was 130/70 mmHg, and her heart rate was 88 beats/min. A high-pitched, decrescendo, grade IV/VI diastolic murmur was audible at the left lower sternal border upon physical examination. Ambulatory blood pressure monitoring indicated elevated daytime and particularly nighttime systolic BP values (152 mmHg and 172 mmHg, respectively). An electrocardiogram showed the voltage criteria for left ventricular (LV) hypertrophy.

**Figure 1:**
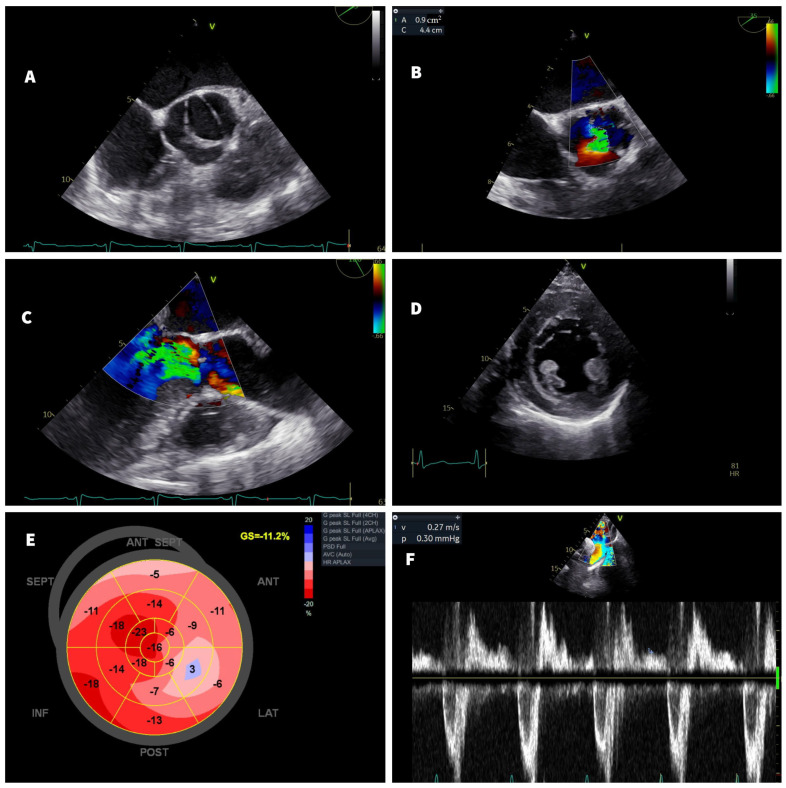
Transthoracic and transesophageal echocardiography. (**A**,**B**) Mid-esophageal short-axis view- bicuspid aortic valve with the fusion of the right and noncoronary cusp and color Doppler of the aortic valve with severe regurgitation. (**C**) Mid-esophageal long-axis view- color Doppler showing severe eccentric aortic regurgitation jet, with the jet width/left ventricle outflow tract (LVOT) diameter >90%. (**D**) Parasternal short-axis view- severe concentric left ventricular hypertrophy. (**E**) Bull’s-eye plot of longitudinal strain showing the reduced global longitudinal strain. (**F**) Pulsed Doppler recording within the descending aorta demonstrates flow reversal throughout the diastole, with an end diastolic flow velocity of 27 cm/s.

Transthoracic echocardiography (TTE) showed a bicuspid aortic valve with grade II calcification and an eccentric jet of severe AR directed along the anterior mitral valve leaflet. Transesophageal echocardiography further detailed the bicuspid aortic valve, with a raphe formed by the fusion between the right coronary cusp and the noncoronary cusp. The diameter of the ascending aorta was 41 mm. The LV was dilated (EDV = 131 mL/m^2^ and ESV = 72 mL/m^2^), with severe symmetric hypertrophy. The left ventricle ejection fraction (LVEF) was mildly reduced (53%) but with a reduced global longitudinal strain (GLS = −11.2%) and diastolic dysfunction grade III (Figure 1).

Laboratory tests revealed increased serum values of N-terminal pro-b-type natriuretic peptide (NT-pro-BNP = 15,449 pg/mL) with raised levels of C-reactive protein (CRP) (39.03 mg/L, normal range 0–5 mg/L). The liver, renal, and thyroid functions were not affected, and no sign of bacterial infection was present.

**Figure 2:**
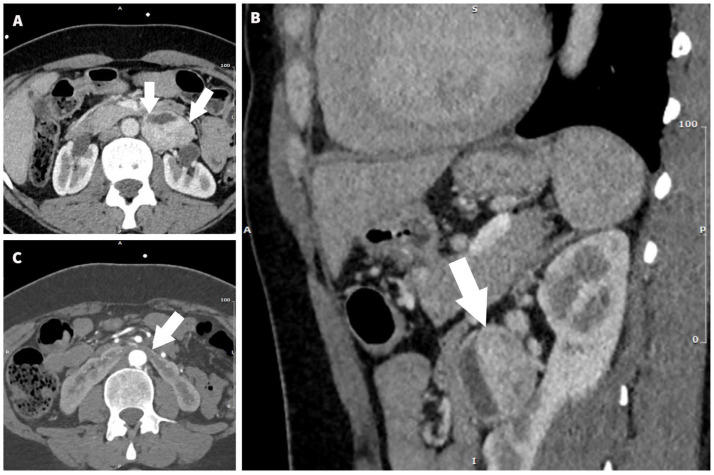
Abdominal CT with contrast shows an inhomogeneous, well-delimited solid mass in the retroperitoneal cavity with areas of necrosis and with strong arterial enhancement (white arrows). (**A**) Axial section. (**B**) Sagittal oblique MPR reconstruction. (**C**) Horseshoe kidney, with symphysis at the level of the lower pole of the right kidney and left kidney.

A CT angiography was performed to investigate the secondary causes of arterial hypertension and associated congenital abnormalities. The CT showed a 36/50/44 mm retroperitoneal space-occupying solid lesion in the left paraaortic space, next to the L1 and L2 vertebral bodies and left renal hilum. After contrast administration, the tumor displayed a similar aspect, with avid enhancement due to hypervascularization, a description suggestive of a paraganglioma (Figure 2). The CT also confirmed the bicuspid aortic valve and the enlargement of the ascending aorta (maximum diameter = 40 mm).

After twenty-four hours, the urinary normetanephrine was increased to 2227 mcg (normal value of <549.5 mcg), with a normal value of metanephrine and elevated plasma chromogranin of 173 mcg/L (normal range 27–94 mcg/L).

Based on these examination results, we considered that the cause of uncontrolled hypertension and associated symptoms was the retroperitoneal tumor, with imaging characteristics that suggested a paraganglioma. A multidisciplinary team of cardiology, cardiac surgery, anesthesiology, urology, and endocrinology specialists concluded that paraganglioma resection should be completed first, followed by an aortic valve replacement if necessary. The reason behind this approach was the high risk for hypertensive crisis and mortality during cardiac surgery due to the potential for unpredictable catecholamine release intra- and perioperatively.

**Figure 3:**
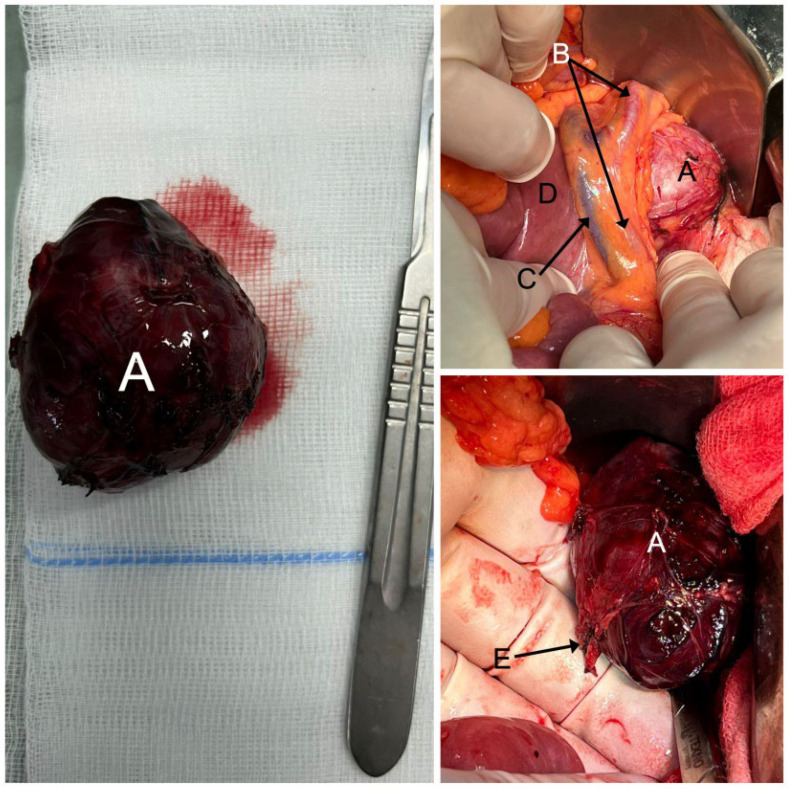
Intraoperative images: (**A**) retroperitoneal paraganglioma; (**B**) left colic artery; (**C**) inferior mesenteric vein; (**D**) Treitz angle; (**E**) main arterial pedicle—branch of the inferior mesenteric artery.

Adequate preoperative management was crucial before resection of the paraganglioma to control the BP and heart rate values during surgery, to avoid complications, and to obtain better results. The alpha-blocker dose was increased, followed by an adjustment of the beta-blocker dose to suppress sympathetic activity and reduce surgical mortality. After the BP and heart rate control, the patient was admitted to the general surgery ward, where the resection of the left retroperitoneal lesion was successfully performed (Figure 3).

**Figure 4:**
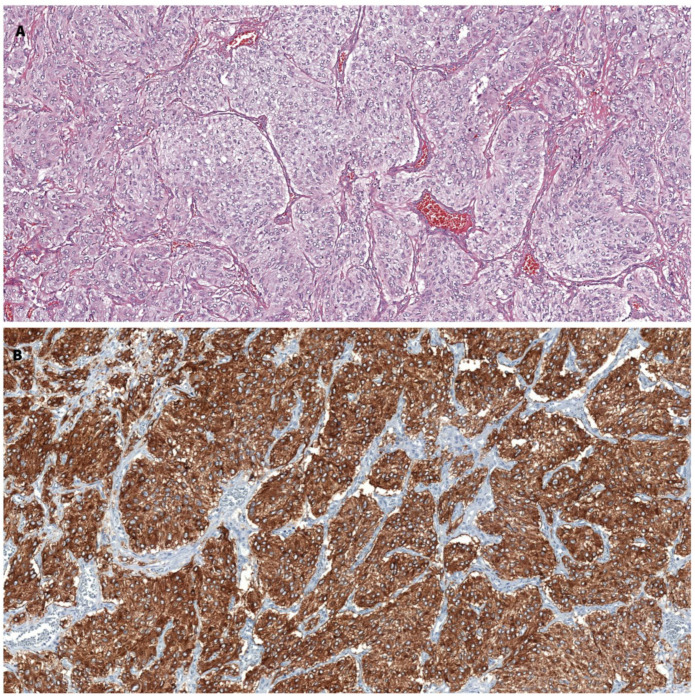
Histopathological examination. (**A**) Hematoxylin–eosin staining, ×100: nests and cords of epithelioid cells separated by a thin fibrovascular stroma. (**B**) Synaptophysin immunohistochemical stain, ×100: tumor cells have a diffuse strong cytoplasmatic positivity.

The macroscopic paraganglioma examination revealed a 55/45/35 mm nodular encapsulated and heterogeneous tumor with hemorrhagic areas. Microscopic examination exhibited a proliferation comprised of epithelioid cells with round to oval nuclei and amphophilic cytoplasm arranged in nests with focal spindle-shaped cells. Immunohistochemical stains showed strong and diffuse positivity for synaptophysin and a Ki67 proliferation index lower than 1%, with a negative S100 stain. The histopathological aspect, together with the immunohistochemical study and the clinical presentation, confirmed the diagnosis of a paraganglioma (Figure 4).

The patient had no post-surgical complications and was discharged 4 days after surgery.

**Figure 5:**
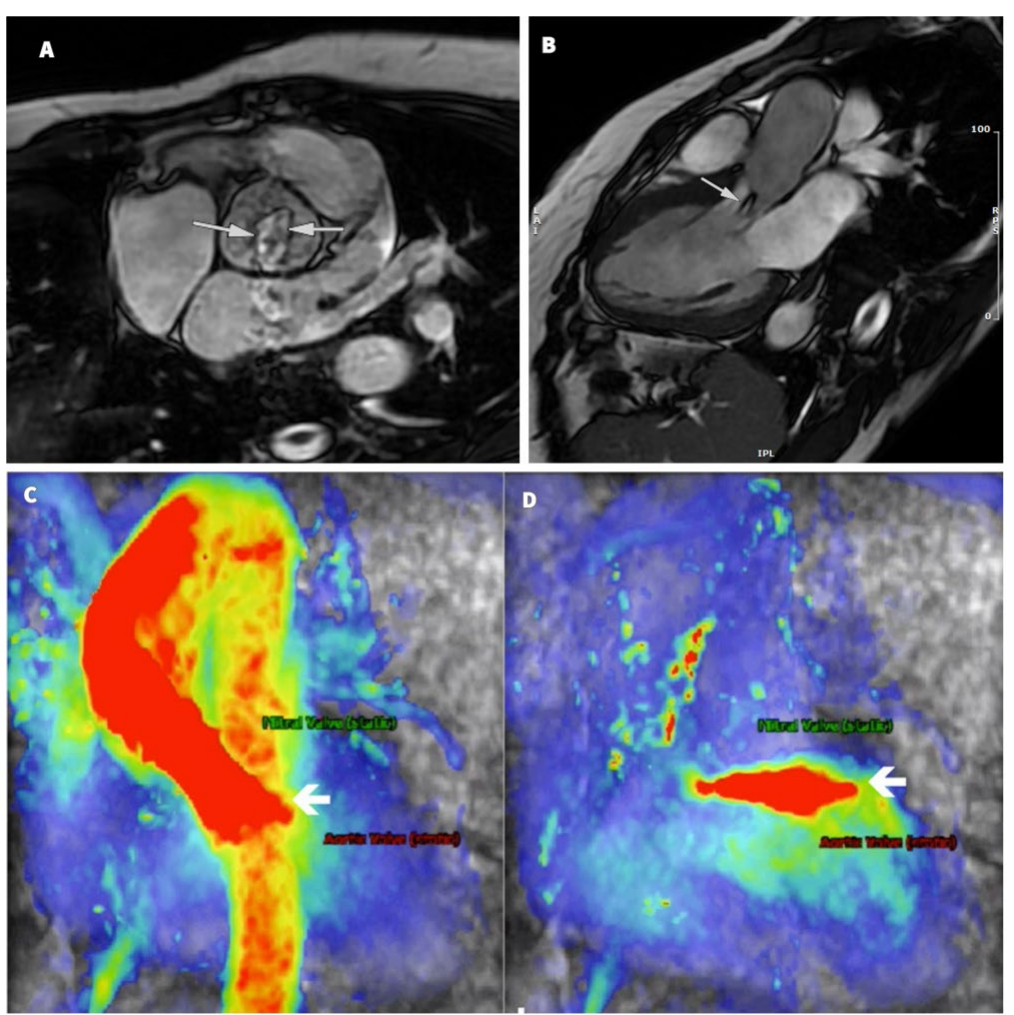
Cardiac magnetic resonance imaging: (**A**) Cine Fiesta sequence in the transverse plane: bicuspid aortic valve type 0, lateral (arrows). (**B**) Cine Fiesta sequence 3: aortic insufficiency jet (arrow). (**C**,**D**) Sequence 4D flow: aorta in systole (**C**), blood flow coded in red (arrow), and in diastole (**D**), highlighting a jet of severe aortic valvular insufficiency with oblique orientation (arrow).

At the six-month follow-up, her BP values were well controlled under treatment with angiotensin II receptor blockers, calcium channel blockers, beta-blockers, and mineralocorticoid receptor blockers, allowing for a gradual reduction in dosage and cessation of alpha-1 adrenergic receptor antagonists and centrally active drugs. The echocardiography reconfirmed the severity of the aortic regurgitation, but a recovery of systolic function with improvement in terms of the LVEF was noted (59%). However, there might be residual subclinical impairment, as demonstrated by the reduced GLS (−13%). The LV diameters were within the normal range, but the LV volumes were increased. The main improvement after the paraganglioma removal was clinical, with the patient relating a significantly improved exercise tolerance, confirmed by an exercise stress test. The serum values of NT-pro-BNP were also normal (190 pg/mL). We performed a cardiac magnetic resonance (CMR) scan that showed severe AR with a regurgitation fraction of 45.7%. The LV was dilated and hypertrophied, with the LVEF = 61% but without signs of cardiac fibrosis, inflammation, or ischemia (Figure 5). The patient also received an abdominal and pelvic contrast-enhanced MRI that did not show residual or recurrent lesions.

Corroborating these results, in the face of an asymptomatic patient with severe AR, preserved LVEF, and an LV end systolic diameter <50 mm, a decision to delay the aortic valve replacement was made. Even if this patient has not yet fulfilled the guidelines indicated for aortic valve replacement, she is at risk of decompensation, so a close follow-up with regular visits to assess her symptoms, NT-pro-BNP values, and echocardiography was decided.

## 3. Discussion

A PPGL diagnostic workup may be hampered by nonspecific clinical symptoms, especially in patients presenting with cardiac symptoms. To our knowledge, this is the first reported case of combined severe aortic regurgitation and paraganglioma, a unique diagnostic challenge that required multidisciplinary care.

### 3.1. Secondary Arterial Hypertension, Catecholamine Crisis, and Acute Aortic Dissection

In our case, the patient presented with persistently elevated BP values unresponsive to treatment, which led to further testing that revealed PPGL as a secondary cause for resistant uncontrolled hypertension. The suspicion of secondary hypertension was raised 17 years ago, but the patient did not undergo any investigation, which delayed the diagnosis of a catecholamine-releasing tumor.

Catecholamine excessive release caused by chromaffin cell tumors produces chronic hypertension or a paroxysmal elevation of BP associated with symptoms of hyperactivity of the sympathetic system, such as the classic triad of profuse sweating, palpitations, and headaches [7]. PPGLs are responsible for a minority of cases of secondary endocrine hypertension and are accountable for only 0.1–0.6% of hypertensive patients. In functional PPGLs, catecholamines are produced and metabolized to their O-methylated derivates within the tumor tissue: dopamine to methoxytyramine, norepinephrine to normetanephrine, and epinephrine to metanephrine. Their release in the plasma and urine is the basis for a biochemical diagnosis in these patients. The conversion of active catecholamines into inactive metabolites within the tumor delays the diagnosis, as cardiac complications and hypertension are produced when the active catecholamines are released [8].

Epinephrine, norepinephrine, and dopamine are the main catecholamines responsible for cardiovascular complications. Their effects on arterial BP values depend on the activation of specific endothelium receptors: norepinephrine activates α-receptors and induces vasoconstriction and volume contraction, increasing the peripheral vascular resistance and consequently raising both the systolic blood pressure (SBP) and diastolic blood pressure (DBP) values, while epinephrine acts additionally on the β2-receptors, producing vasodilation in the peripheric skeletal muscles and increasing the SBP, with no significant effects on the DBP values [9]. Noradrenergic-secreting PPGLs are more frequently associated with sustained hypertension, while epinephrine-secreting tumors induce paroxysmal symptoms. Dopamine-releasing tumors are rare; patients present with normal or low blood pressure values [8,9]. The noradrenergic subtype of PPGLs has been associated with increased target organ damage to the heart and vessels and impaired diurnal variability of blood pressure values [10].

A massive surge of catecholamines can lead to the development of a life-threatening complication of PPGLs, known as a catecholamine crisis. The excessive serum concentration of active sympathetic amines produces unstable BP values and cardiovascular collapse in the form of heart failure and pulmonary edema, increasing the mortality risk in patients with undiagnosed PPGLs [11,12]. This hypertensive crisis may be triggered by psychological stressors; severe pain; situations related to anesthesia, such as induction or intubation; mechanical stimuli; or drugs that interact with adrenergic signaling, such as dopamine receptor antagonists, antidepressants, beta-blockers, or sympathomimetics [4].

The abrupt and severe elevation of BP values caused by catecholamines-secreting tumors can lead to a rare life-threatening complication that manifests as acute aortic dissection. The association between paragangliomas and acute aortic dissection is very rare, with only two cases being described in the literature [13,14]. The use of multimodality imaging is essential for the confirmation of acute aortic syndrome [15]. CT is recommended as the first step imaging method, due to its wide availability, rapidity in performing, and high specificity and sensitivity. It includes a full assessment of the aorta anatomy and identifies the presence of complications. In hemodynamically unstable patients, transthoracic echocardiography and transesophageal echocardiography are used for the evaluation of the ascending aorta and the identification of cardiac complications, such as AR or pericardial effusion. MRI is recommended mainly for the follow-up of patients with uncertain diagnosis or in stable patients with contraindication for the iodinated contrast [16].

### 3.2. Catecholamine-Induced Cardiomyopathy

Our patient’s LV was dilated and hypertrophied due to multiple concomitant causes that included aortic regurgitation and long-standing secondary hypertension, as well as a direct effect of the catecholamines excess, causing elevated serum levels of NT-pro-BNP, as a marker of the myocardial strain and remodeling. The improvement in the echocardiographic parameters and NT-pro-BNP levels after the tumor removal indicates a direct hemodynamic effect of catecholamines on cardiac function and its dimensions.

High levels of circulating catecholamines have been associated with specific cardiomyopathies, such as dilated cardiomyopathy (DCM), hypertrophic cardiomyopathy (HCM), myocarditis, and Takotsubo cardiomyopathy [17]. LV hypertrophy and remodeling are found in 25–40% of patients with PPGLs as a result of both pressure overload and the direct effect of catecholamines on myocardial protein synthesis [9,18]. An echocardiography-based study of 81 patients with PPGLs suggested that the excessive release of catecholamines leads to LV hypertrophy and impairs systolic and diastolic LV functions, alterations independent of the blood pressure values. After the surgical removal of the lesion, these changes in the LV structure, as well as in the systolic and diastolic LV function, were improved [19]. In another two-dimensional speckle tracking echocardiography study, the excessive release of norepinephrine in the PPGLs group was associated with an elevated LV mass and BP levels. Regarding LV function, although the EF was similar in patients with PPGLs and control patients, the LV global longitudinal strain (GLS) was decreased in patients with catecholamine-releasing tumors [20]. Comparative to subjects with essential hypertension, with similar BP levels, patients with pheochromocytoma displayed lower GLS values. These results suggest that catecholamines impact the ventricular geometry and alter the systolic function at the subclinical level [20,21].

Interestingly, PPGLs have been reported to cause primary cardiomyopathy characterized by dilated chambers and cardiac hypertrophy in normotensive patients. The molecular mechanisms responsible for direct myocardial damage are an intracellular calcium overload, oxidative stress activation with the generation of reactive oxygen species (ROS), and increased membrane permeability. An excessive amount of catecholamines can also induce functional myocardial ischemia due to the imbalance between the demand and supply of oxygen regarding increased cardiac contractility, coronary arterial vasospasm, and platelet aggregability [22].

Since catecholamines can produce both acute and chronic myocardial dysfunction, the clinical presentation is similar to patients with idiopathic dilated cardiomyopathy, and it varies from congestive heart failure to cardiogenic shock [23]. Thus, in patients with severe heart failure who undergo cardiac transplantation, PPGLs are included in the causes of reversible cardiomyopathy that must be excluded [24].

Compared to hypertensive cardiac disease, catecholamine-induced cardiomyopathy has different and more extensive alterations. In cardiac magnetic resonance, a pattern of “active catecholamine myocarditis” is described, characterized by impaired myocardial systolic and diastolic LV functions, areas of myocarditis, and diffuse and focal fibrosis. After PPGL removal, although the EF may improve, the persistence of diffuse and local fibrosis, as well as systolic and diastolic strain impairment, suggests irreversible subclinical effects of catecholamines in myocardial tissue [25].

### 3.3. Arrhythmias

PPGLs are associated with atrial tachyarrhythmias, particularly sinus tachycardia, paroxysmal supraventricular tachycardia, multifocal atrial tachycardia, and junctional ectopic tachycardia [9,26,27]. However, the association between paraganglioma and AVNRT as a type of paroxysmal supraventricular tachycardia is uncommon. The excess of catecholamines released by the paraganglioma can be involved in the manifestation of AVNRT due to frequent atrial premature beats.

Among patients diagnosed with pheochromocytoma, 50–70% complained of palpitations [9]. In the atria, catecholamines act on β-adrenoreceptors and promote the development of sinus tachycardia, whereas, in the ventricles, the activation of β-adrenoreceptors promotes ventricular arrhythmogenesis and prolongs the QT interval in predisposed subjects, increasing the risk of torsade des pointes ventricular tachycardia [2,28,29]. In one group of patients with cardiac complications caused by pheochromocytoma, the most commonly encountered arrhythmia was atrial fibrillation, followed by supraventricular tachycardia and ventricular tachycardia as torsade des pointes [29].

The excess of catecholamines was also associated with bradyarrhythmia, including sinus node dysfunction and atrioventricular blocks. The physiopathological mechanism involves a baroreceptor reflex response to the rapid increase in blood pressure and the desensitization of adrenergic receptors due to prolonged stimulation, which leads to the predominance of vagal activation [29,30].

PPGLs may present with arrhythmias that are resistant to conventional therapies due to the surge of catecholamines. In a case of pheochromocytoma and left ventricular non-compaction, sustained ventricular tachycardia was resistant to conventional therapy after direct cardioversion shock administration and antiarrhythmic drugs. After tumor removal, the patient did not experience any symptoms, and the implanted cardioverter-defibrillator interrogation showed no further episodes of ventricular tachycardia [31]. Sustained ventricular tachycardia might have been induced by the catecholamine hyperstimulation of β-receptors, suggesting the essential role of β-blockers as antiarrhythmic drugs in patients with pheochromocytoma [32].

### 3.4. Valvular Heart Disease

It is unclear the period in which the paraganglioma started developing within our patient. There may be a causal connection between the paraganglioma and the subsequent development of AR. The hemodynamic impact of PPGL could have aggravated the AR, which was clinically insignificant in the past. Since the patient had no cardiovascular symptoms and had a normal exercise tolerance in the previous years, we carefully supposed that the AR was worsened by the hemodynamically significant paraganglioma.

Compared to the general population, individuals with a bicuspid aortic valve have an increased risk for surgery of the aortic valve and ascending aorta at a younger age. In a cohort of patients with a bicuspid aortic valve, aged older than 30 years, the severity of aortic stenosis and the severity of aortic regurgitation were predictors of cardiac complications [33].

The current guidelines recommend surgery for asymptomatic patients with severe AR and the impairment of LV function or LV enlargement, as well as for symptomatic patients irrespective of a LVEF [34]. Therefore, an objective functional capacity assessment is also recommended to guide decision-making. Patients with AR may demonstrate a hyperdynamic LV in the early stages, before systolic function impairment in more advanced, severe AR, with a poor contractile reserve demonstrated during exercise testing [35].

In asymptomatic patients with severe AR, further echocardiographic and CMR parameters can predict a worse prognosis, suggesting the need for aortic valve surgery in additional circumstances. LV enlargement, evidenced by a LV end systolic volume index (LVESVi) greater than 45 mL/m^2^, increased the mortality risk in a cohort of 492 asymptomatic chronic moderately severe to severe AR. In the same cohort, a LVEF lower than 60% was associated with all-cause mortality [36]. The impairment of the baseline longitudinal or circumferential strain of the LV was a predictor of the need for surgery during follow-up in asymptomatic patients with a LVEF > 50% and moderate-to-severe or severe AR [37]. Furthermore, a regurgitation fraction > 33%, LVEDV > 246 mL, and the presence of a myocardial scar quantified by CMR showed the best prediction of progression to surgery in patients with echocardiographic moderate or severe AR [38].

## 4. Conclusions

Paraganglioma is a rare chromaffin cell tumor similar in manifestation to pheochromocytoma. The long-standing release of catecholamines leads to several cardiovascular complications, including uncontrolled arterial hypertension. We reported a case of a late-diagnosed paraganglioma in a young woman with secondary hypertension who presented with severe aortic regurgitation. This case highlights the importance of multimodality imaging using computed tomography and magnetic resonance imaging in the diagnosis and management of two challenging cardiovascular events. Paraganglioma resection was essential to decide between aortic valve intervention versus a watchful waiting strategy. The successful removal of the tumor ameliorated the patient’s exercise tolerance and improved the cardiac hemodynamic parameters, and a close follow-up of aortic regurgitation was recommended.

## Data Availability

Not applicable.
